# P05-14 Effects of a 12-weeks aquatic fitness program in women with osteoarthritis

**DOI:** 10.1093/eurpub/ckac095.081

**Published:** 2022-08-29

**Authors:** Pedro Morouço

**Affiliations:** Polytechnic Institute of Leiria, Leiria, Portugal

**Keywords:** cartilage disorders, ageing, conditioning, strngth

## Abstract

**Background:**

As a major public health concern, there is a high association between aging and obesity, nutritional deficiencies and physical (in)activity. Thus, diseases related to cartilage are on the list of main concerns of the WHO, assuming the prevention of degeneration of articular cartilage as an important issue for which there are few effective solutions. It is imperative to find preventive strategies that can reduce the incidence of chronic osteoarthritis.

**Methods:**

Eleven women (58.1±3.3 years-old) diagnosed with knee osteoarthritis (KOA) enrolled in tri-weekly aquatic fitness 45' sessions, for 12 weeks. Taking advantage of the physical properties of the water for increasing the load, a gradual use of the extension of the levers was defined. The warm-up focused on body alignment, joint mobility and breathing. The fundamental part was based on exercises that combine the cardiorespiratory component with the strength component, promoting a superior range of motion. At the end of each session there was a progressive decrease in load, alternating body segments. Before and after the 12 weeks they performed the Senior Fitness Test, hand-grip strength and body measures. All participants were volunteer, informed consent was obtained and all procedures were in accordance to Helsinki Declaration. Sessions were instructed by a CSCS®.

**Results:**

Significant and meaningful improvements were observed in lower body strength (p > 0.001; d = 1.10), lower body flexibility (p > 0.001; d = 2.88), aerobic endurance (p > 0.001; d = 0.95), dynamic balance (p > 0.001; d = 1.22) and hand grip strength (p > 0.001; d = 1.56). Significant, but moderate improvements were observed in body mass (p = 0.034; d = 0.56) and waist circumference (p = 0.041; d = 0.66).

**Conclusions:**

Aquatic fitness induced extensive benefits in women conditioning, suggesting that this activity is able to promote an increase in life quality, even if KOA is diagnosed. This program aims to be a tool for implementing healthy behaviour, based on a physical exercise program to people with KOA. With a demographic trend towards an aging population, today society has dragged itself into a vicious cycle to the proven relationship between OA and obesity, and the increasing prevalence of both. To contribute to solving these problems, it is mandatory to have interdisciplinary perspectives that promote a motivating and lasting activity.

